# The monoclonal antibody AZD5148 confers broad protection against TcdB-diverse *Clostridioides difficile* strains in mice

**DOI:** 10.1371/journal.ppat.1013651

**Published:** 2025-11-03

**Authors:** F. Christopher Peritore-Galve, Heather K. Kroh, John A. Shupe, Alyssa G. Ehni, Rubén Cano Rodríguez, Shannon L. Kordus, M. Kay Washington, Reece J. Knippel, Ann Marie Stanley, Adam Gamson, Christine Tkaczyk, D. Borden Lacy

**Affiliations:** 1 Department of Pathology, Microbiology, and Immunology, Vanderbilt University Medical Center, Nashville, Tennessee, United States of America; 2 Early Vaccines and Immune Therapies, BioPharmaceuticals R&D, AstraZeneca, Gaithersburg, Maryland, United States of America; 3 Department of Veterans Affairs, Tennessee Valley Healthcare System, Nashville, Tennessee, United States of America; Texas A&M University, UNITED STATES OF AMERICA

## Abstract

*Clostridioides difficile* is the leading cause of antibiotic-associated intestinal infections. The pathogenesis of *C. difficile* infection (CDI) is driven by two protein exotoxins, TcdA and TcdB. The TcdB-targeting monoclonal antibody (mAb) bezlotoxumab (Zinplava) was indicated to reduce CDI recurrence in patients 18 years of age or older who are receiving antibacterial drug treatment for CDI and are at high risk for CDI recurrence. However, Zinplava has recently been discontinued, underscoring the need for additional therapeutics. AZD5148 is a humanized anti-TcdB mAb that neutralizes toxin activity by blocking the delivery of the enzymatic glucosyltransferase domain (GTD) into host cells. TcdB sequence variation influences receptor tropism and substrate specificity, with three major subtypes—TcdB1, TcdB2, and TcdB3—representing the dominant diversity among clinical isolates. In this study, we evaluated the protective efficacy of AZD5148 *in vitro* and *in vivo* against clinically relevant *C. difficile* strains expressing these three dominant TcdB subtypes. AZD5148 potently neutralized TcdB1 and TcdB2 *in vitro*, with EC_50_ values 1,000- to 14,000-fold lower than those of bezlotoxumab. In a mouse CDI model induced by TcdB1- or TcdB2-expressing strains, AZD5148 provided robust protection against weight loss and mortality at significantly lower doses than bezlotoxumab. Furthermore, the addition of the anti-TcdA mAb, PA50, provided no additional protective benefit. Although AZD5148 did not neutralize TcdB3 *in vitro*, it significantly reduced intestinal edema and inflammatory cell infiltration in mice infected with a TcdB3-producing strain. These findings demonstrate that AZD5148 offers broad-spectrum protection against *C. difficile* strains and retains *in vivo* efficacy even in the absence of *in vitro* neutralization. Its distinct mechanism of action and superior potency compared to bezlotoxumab support its continued development as a promising therapeutic candidate for the prevention of a first CDI episode and prevention of recurrence.

## Author summary

*C. difficile* is the leading cause of antibiotic-associated diarrhea and intestinal infections, causing a significant burden on patients and the healthcare system. The disease is driven by two large protein toxins: toxin A (TcdA) and toxin B (TcdB), which damage host cells and trigger inflammation. Antibiotics are the standard of care to treat infection, but they disrupt the gut microbiome, leaving patients at risk for recurrence. In contrast, monoclonal antibodies (mAbs) that neutralize these toxins offer a promising therapeutic strategy, especially for high-risk patients. Bezlotoxumab (Zinplava), an FDA-approved mAb targeting TcdB, was effective in reducing recurrence but has recently been discontinued. We previously identified AZD5148, a humanized mAb that blocks the delivery of the toxic TcdB glucosyltransferase enzyme into host cells by targeting a conserved region of the enzymatic domain. This contrasts with bezlotoxumab, which binds to the TcdB combined repetitive oligopeptide sequence (CROPS) domain and functions by interfering with receptor binding. In this study, we demonstrate that AZD5148 protects mice from infection with multiple clinically relevant *C. difficile* strains that produce the three most prevalent TcdB sequence variants. Notably, AZD5148 was effective at lower doses than bezlotoxumab and provided protection, even against a strain that produced a TcdB variant that it could not neutralize *in vitro*. These findings highlight the potential of AZD5148 as a broadly protective biotherapeutic to prevent and treat *C. difficile* infection.

## Introduction

*Clostridioides difficile* infection (CDI) is the leading cause of antibiotic-associated intestinal infections, with 462,100 cases and 12,800 deaths reported in the U.S. alone in 2017 [1,2]. *C. difficile* is a Gram-positive, spore-forming anaerobe transmitted via the fecal-oral route. Once in the gut, the spores exploit antibiotic-induced dysbiosis to germinate and proliferate [3]. This outgrowth in the colon can result in a spectrum of disease severity, ranging from mild diarrhea to severe complications like pseudomembranous colitis, toxic megacolon, sepsis, and death. Current clinical guidelines recommend fidaxomicin or vancomycin as the standard of care treatment for CDI [4]. While these antibiotics are effective in achieving initial clinical cure, they perpetuate gut dysbiosis, leaving patients vulnerable to recurrent CDI [1,3,5].

CDI pathogenesis is driven by the two large protein exotoxins, TcdA and TcdB [6]. These toxins bind to host cell receptors and are internalized via endocytosis. Endosomal acidification triggers toxin conformational changes that facilitate pore formation and translocation of the glucosyltransferase domain (GTD) into the host cytosol. The GTD then irreversibly inactivates Rho- or Ras-family GTPases, disrupting the cytoskeleton, inducing proinflammatory cytokine production, and causing cell death [7]. Mouse models of infection with isogenic toxin knockout strains show that while both toxins may contribute to pathogenesis, TcdB alone is sufficient to cause severe disease [8–11]. Clinical observations similarly indicate that patients infected with TcdA^-^ TcdB^+^ strains can experience the full range of CDI [12].

Current alternative therapies, such as fecal microbiota transplantation (FMT) and monoclonal antibodies, are being used alongside antibiotics to reduce the risk of recurrent CDI [13–16]. Bezlotoxumab (Zinplava), a TcdB-neutralizing monoclonal antibody (mAb), was FDA-approved to prevent recurrent CDI in high-risk patients, but its production was discontinued in January 2025 [16,17].

CDI, once considered predominantly a nosocomial infection, has seen a significant rise in community-acquired cases, with 50% of reported CDIs in 2017 occurring outside of healthcare facilities [1,18]. This shift has been attributed, in part, to the emergence and diversification of new epidemic *C. difficile* strains, such as the NAP1/BI/ribotype (RT) 027 strain R20291, which was prevalent in North America during the early 2000s before its decline in the 2010s [1,19].

Ongoing global surveillance have led us to appreciate that strains can also be classified according to which TcdB sequence variant they encode [19–21]. Three TcdB subtypes are most prevalent in clinical isolates: TcdB1, which is the most common in clinical isolates and laboratory strains like VPI 10463 and 630; TcdB2, associated with epidemic RT027 strains like R20291; and TcdB3, which is found in RT017 strains like M68, which are endemic to Asia [19,21,22]. These three toxin subtypes differ in the cellular receptors they interact with and the GTPase substrates they glucosylate [6,23]. Bezlotoxumab blocks TcdB binding to the chondroitin sulfate proteoglycan 4 (CSPG4) receptor, but is less effective against different variants due to differences in receptor binding [23,24].

Given the variability in TcdB sequences and receptor interactions, there is a pressing need for therapeutics that target conserved, neutralizing epitopes across TcdB variants. We previously described PA41 (now called AZD5148), a humanized mAb that binds a highly conserved TcdB GTD epitope and prevents the delivery of the GTD enzymatic cargo into the host cytoplasm [25,26]. In concept, this mechanism that acts downstream of receptor binding may offer broader efficacy than bezlotoxumab, particularly across TcdB variants with differing receptor tropisms. To evaluate this, we first tested whether AZD5148 alone could confer protection in a CDI mouse model with the epidemic strain R20291. Seeing that it did, we then tested AZD5148 against the highly virulent, TcdB1-producing strain VPI 10463 and the TcdB3-producing strain M68, which harbors a mutation in the AZD5148 epitope. Although AZD5148 did not neutralize TcdB3 *in vitro*, it still conferred *in vivo* protection against edema and inflammation. Here, we discuss these findings in the context of our current understanding of the TcdB mechanism of action, strain-specific differences in pathogenesis, and the therapeutic potential of AZD5148.

## Results

### AZD5148 neutralizes TcdB1 and TcdB2 but not TcdB3 *in vitro*

**Fig 1:**
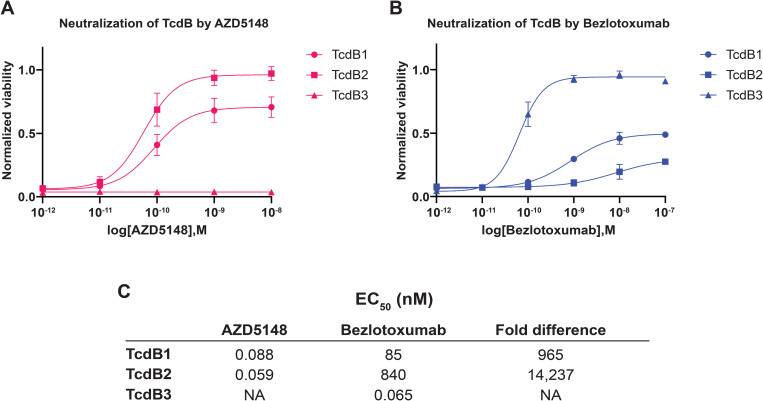
AZD5148 potently neutralizes TcdB1 and TcdB2 but not TcdB3 *in vitro.* **(A-B)** *In vitro* neutralization of 30 fM TcdB1, TcdB2, or TcdB3 by AZD5148 **(A)** or bezlotoxumab **(B)** in Vero cells. Data points represent the mean of three replicates; error bars indicate the standard error of the mean. **(C)** Half-maximal effective concentration (EC_50_) values for each mAb against the three TcdB subtypes were calculated from the neutralization curves shown in panels A and B.

Previous studies using strain culture supernatants indicated that AZD5148 broadly neutralizes TcdB, demonstrating superior efficacy compared to bezlotoxumab, except against toxins from RT017 strains [25]. Since the presence of other secreted proteins could not be excluded in those previous experiments, we evaluated AZD5148 and bezlotoxumab neutralization *in vitro* using purified proteins from the three most prevalent toxin subtypes. In toxin neutralization assays conducted in Vero cells, which are highly sensitive to TcdB [27], AZD5148 neutralized TcdB1 and TcdB2 with EC_50_ values of 88 pM and 59 pM, respectively (Fig 1A), representing 965-fold and 14,237-fold greater potency than bezlotoxumab (Fig 1B). However, AZD5148 did not neutralize TcdB3, while bezlotoxumab retained activity with an EC_50_ of 65 pM (Fig 1C). These results demonstrate that the amino acid variation in the epitopes targeted by AZD5148 and bezlotoxumab plays a critical role in their ability to neutralize diverse TcdB subtypes.

### Prophylactic AZD5148 administration protects mice against CDI induced by the TcdB2-producing R20291 strain

**Fig 2:**
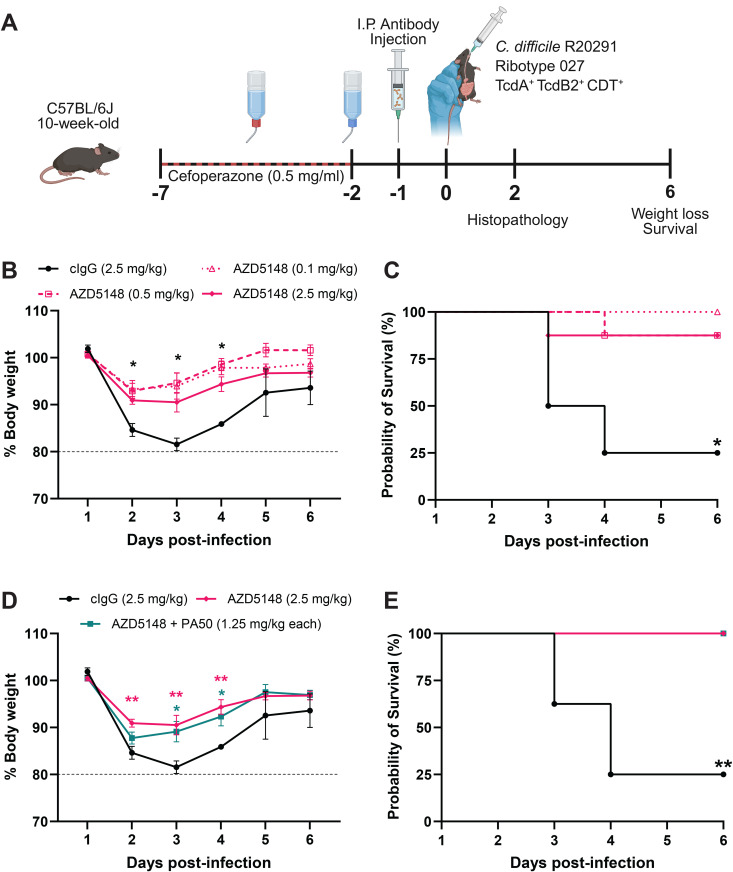
AZD5148 protects against severe disease caused by the RT027 strain R20291. **(A)** Schematic of experimental design made using BioRender. **(B)** Percent body weight relative to baseline in mice pre-treated with 0.1, 0.5, or 2.5 mg/kg AZD5148 or control IgG (cIgG) before infection with *C. difficile* R20291 (n = 4, 8, 8, 8, respectively). **(C)** Six-day survival analysis corresponding to the treatment groups in panel B. **(D)** Percent body weight in mice pre-treated with cIgG, 2.5 mg/kg AZD5148, or a combination of 1.25 mg/kg AZD5148 and 1.25 mg/kg PA50 (n = 8 per group). The cIgG and 2.5 mg/kg AZD5148 groups are the same as in panel B. Pink asterisks indicate significant differences between AZD5148 and cIgG; teal asterisks indicate differences between AZD5148 + PA50 and cIgG. **(E)** Six-day survival analysis for the treatment groups shown in panel D. Data points represent group means; error bars indicate the standard error of the mean. * *P* < 0.05, ** **P* *< 0.01.

To understand how AZD5148’s neutralizing activity translates to *in vivo* protection, we first evaluated its protective efficacy in a CDI mouse model using the TcdB2-producing R20291 strain. Mice were administered AZD5148 at 0.1, 0.5, or 2.5 milligram/kilogram (mg/kg) or an irrelevant human IgG1 (cIgG) 24 hours before infection (Fig 2A). AZD5148 significantly reduced weight loss between 2- and 4-days post-infection (**p* *< 0.05; Figs 2B and S1A). Mice treated with any dose of AZD5148 lost only 5–10% of their body weight, compared to up to 18% in the cIgG group (Figs 2B and S1A). Furthermore, AZD5148 accelerated weight recovery and improved survival at all doses tested (**p* *= 0.0244, 0.0097, and 0.0163, respectively, for 0.1, 0.5, and 2.5 mg/kg; Fig 2C).

Historically, the efficacy of anti-TcdB mAbs has been evaluated in combination with anti-TcdA mAbs in hamster CDI models, which do not accurately replicate human CDI pathogenesis [17,25,28]. To determine whether the combination of AZD5148 and anti-TcdA mAb PA50 would provide any additional benefit, we compared the efficacy of AZD5148 (2.5 mg/kg) alone to its combination with PA50 (1.25 mg/kg each). Interestingly, AZD5148 monotherapy provided the same survival protection compared to the combination therapy when compared to cIgG (**p* *= 0.0002; Figs 2D, 2E and S1B). While AZD5148 alone significantly reduced weight loss compared to cIgG (**p* *= 0.0054), the combination therapy did not (**p* *= 0.2423; Figs 2D and S1B). These results suggest that the prophylactic administration of AZD5148 alone is effective in preventing severe CDI in mice.

### AZD5148 improves protection compared to bezlotoxumab at lower doses during TcdB2-producing R20291 infection

**Fig 3:**
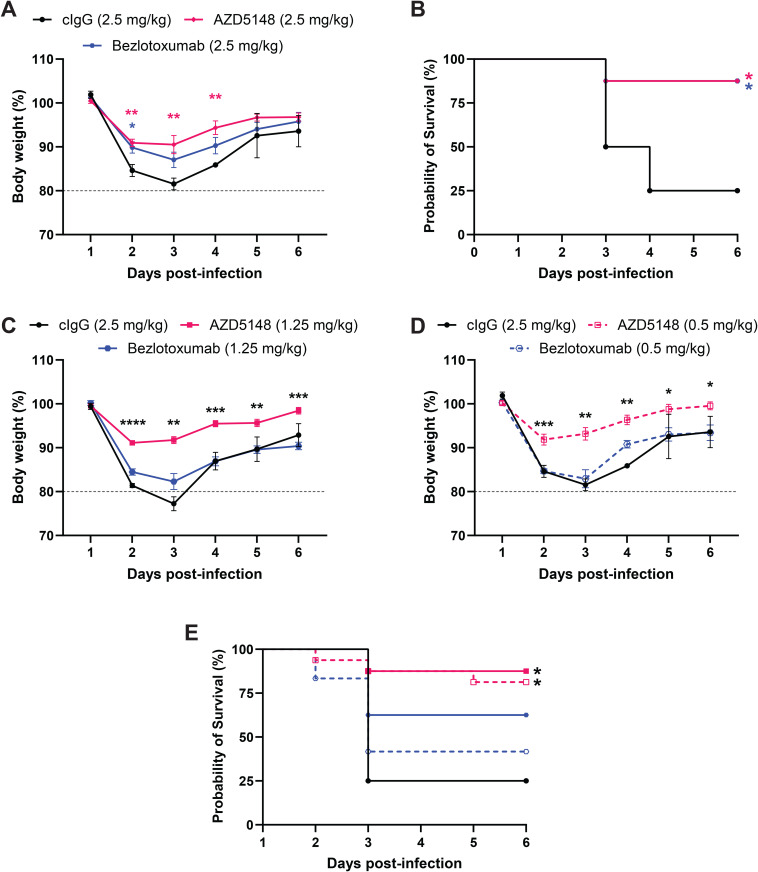
AZD5148 provides superior protection compared to bezlotoxumab at equivalent low doses during R20291 infection. **(A)** Percent body weight relative to baseline in mice pre-treated with cIgG, 2.5 mg/kg AZD5148, or 2.5 mg/kg bezlotoxumab before infection with *C. difficile* R20291 (n = 8 per group). Red asterisks indicate statistically significant differences between AZD5148 and cIgG; blue asterisks indicate differences between bezlotoxumab and cIgG. **(B)** Six-day survival analysis for the treatment groups shown in panel A. **(C)** Percent body weight in mice pre-treated with cIgG, 1.25 mg/kg AZD5148, or 1.25 mg/kg bezlotoxumab (n = 8 per group). Black asterisks denote significant differences between AZD5148 and bezlotoxumab. **(D)** Percent body weight in mice pre-treated with cIgG, 0.5 mg/kg AZD5148, or 0.5 mg/kg bezlotoxumab (n = 8, 16, and 12, respectively). **(C)** Six-day survival analysis for the treatment groups shown in panels C and D. Asterisks indicate statistically significant differences between the AZD5148-treated group and the cIgG group. Data points represent group means; error bars indicate standard error of the mean. * **P* *< 0.05, ** **P* *< 0.01, *** *P* < 0.001, **** **P* *< 0.0001.

Since bezlotoxumab was shown to be clinically effective as a single mAb [16], we next compared the efficacy of AZD5148 with that of bezlotoxumab in the mouse CDI model. Mice were pretreated with 0.5, 1.25, or 2.5 mg/kg of either mAb before infection with R20291. Both anti-TcdB mAbs at 2.5 mg/kg conferred significant improvement in survival compared to cIgG (*p* = 0.0127). However, AZD5148, but not bezlotoxumab, resulted in significant reductions in weight loss and improved survival compared to cIgG at doses of 1.25 and 0.5 mg/kg (*p* = 0.0147 and 0.0108, respectively; Figs 3C–3E and S2).

To assess the effects of AZD5148 or bezlotoxumab on the epithelium, we performed histological analysis two days post-infection in mice treated with 1.25 mg/kg of either mAb. Significant reductions in cecum epithelial injury and colon inflammation were observed for both bezlotoxumab (**p* *= 0.0196) and AZD5148 (*p = *0.0008), compared to cIgG-treated mice. (S3A–S3I Fig).

We also tested whether mAb neutralization affected *C. difficile* colonization burden in stool and measured pro-inflammatory chemokine CXCL1/KC and cytokine IL-6 levels in blood serum and cecum/colon tissues to assess local and systemic responses to infection. Neither mAb impacted *C. difficile* colonization during acute infection (S3J Fig). However, serum and tissue levels of CXCL1/KC and IL-6 were elevated during infection, and both AZD5148 and bezlotoxumab were effective in lowering CXCL1/KC and IL-6 levels in sera (**p* *< 0.0001), but not in tissue (S3K–S3L Fig). These results suggest that AZD5148 provides superior protection compared to bezlotoxumab, particularly at low doses, and significantly reduces weight loss, survival, and inflammatory responses during infection with the TcdB2-producing R20291 strain.

### AZD5148 protects against TcdB1-producing strain VPI 10463

**Fig 4:**
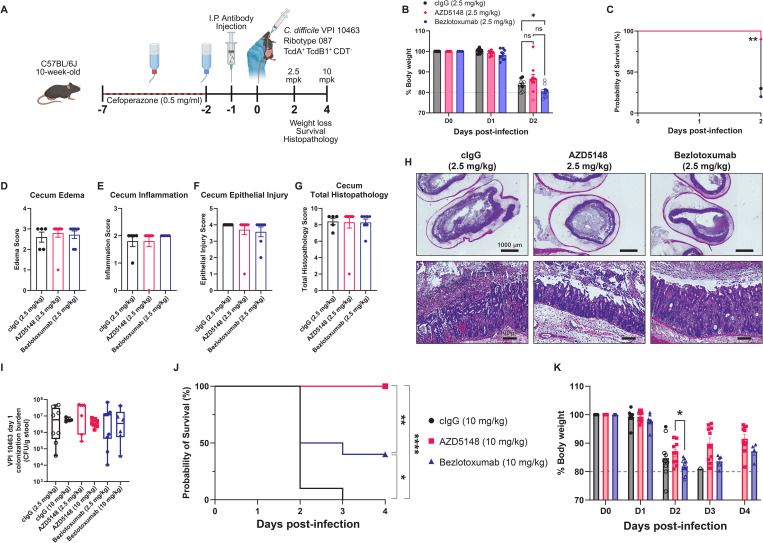
AZD5148 protects against mortality during infection with the TcdB1-producing strain VPI 10463. **(A)** Schematic of experimental design made using BioRender. **(B)** Percent body weight relative to baseline in mice pre-treated with 2.5 mg/kg cIgG, AZD5148, or bezlotoxumab before infection with *C. difficile* VPI 10463 (n = 10 per group). **(C)** Two-day survival analysis for the treatment groups shown in panel B. **(D-G)** Histopathological scoring of cecal tissues collected two days post-infection. **(H)** Representative hematoxylin and eosin (H&E)-stained sections of ceca from each treatment group. **(I)** Four-day survival analysis of mice pre-treated with 10 mg/kg AZD5148, bezlotoxumab, or cIgG (n = 10 per group). **(J)** Percent body weight relative to baseline in mice pre-treated with 10 mg/kg cIgG, AZD5148, or bezlotoxumab prior to infection with *C. difficile* VPI 10463 (n = 10 per group). Data points represent group means; error bars indicate standard error of the mean * *P* < 0.05, ** *P* < 0.01., *** *P* < 0.001, **** **P* *< 0.0001.

We next tested AZD5148 against the highly virulent, TcdB1-producing *C. difficile* strain VPI 10463. Mice were pre-treated with mAbs at 2.5 mg/kg and challenged with 1000 spores (Fig 4A). By two days post-infection, only 30% of cIgG and 20% of bezlotoxumab-treated mice survived, with the remainder succumbing to disease or meeting euthanasia criteria, while AZD5148 treatment resulted in 90% survival (Fig 4B, 4C). Interestingly, all groups exhibited equally severe intestinal pathology (Fig 4D–4H). However, the inability to assess tissue pathology in mice that succumbed to disease may skew the interpretation of these data.

Administration of each mAb at 10 mg/kg resulted in 0% survival for cIgG, 40% survival for bezlotoxumab (*p* = 0.0206 compared to cIgG), and 100% survival for AZD5148 (**p* *< 0.0001 compared to cIgG, *p* = 0.0040 compared to bezlotoxumab; Fig 4I). Due to the severe diarrhea during VPI 10463 infection, we were only able to assess *C. difficile* colonization levels on day one, and no significant differences in colonization were observed between the mAb groups (Fig 4J). AZD5148 also significantly reduced weight loss (**p* *= 0.0066) and accelerated recovery compared to cIgG (Fig 4K). Collectively, AZD5148 provides superior protection at both 10 mg/kg and 2.5 mg/kg compared to bezlotoxumab in the TcdB1-producing VPI 10463 CDI mouse model.

### AZD5148 reduces edema and cellular infiltration induced by the TcdB3-producing strain M68

To evaluate whether AZD5148 provided protection against a TcdB3-producing strain despite poor neutralizing activity *in vitro,* we compared the protective efficacy of AZD5148 and bezlotoxumab in mice infected with *C. difficile* M68, a TcdB3-producing RT017 strain.

**Fig 5:**
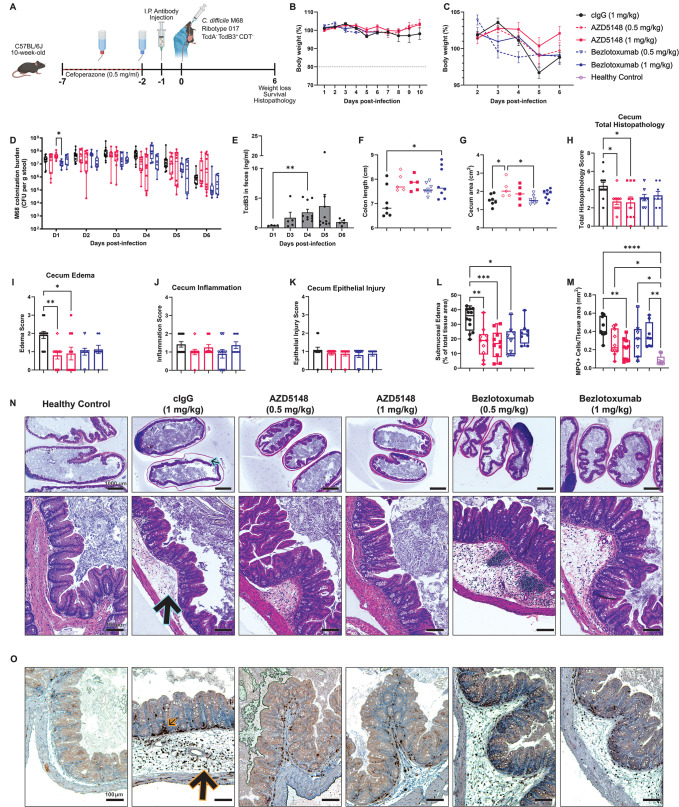
AZD5148 attenuates submucosal edema and inflammation during infection with the TcdB3-producing RT017 strain M68. **(A)** Schematic of experimental design made using BioRender. **(B)** Percent body weight relative to baseline in mice pre-treated with 0.5 or 1 mg/kg AZD5148, bezlotoxumab, or cIgG before infection with M68 (n = 8-17 per group). **(C)** Scaled percent weight change from days two to six post-infection, highlighting 4-8% weight loss. **(D)** Daily *C. difficile* M68 colonization burden in naturally passed stool (n = 8-12 per group). **(E)** TcdB3 concentration in shed stool over the course of M68 infection in cIgG mice (n = 4-10). **(F)** Colon length and **(G)** cecum area measured at day 6 post-infection (n = 5-8 per group). **(H-K)** Histopathological scoring of cecal tissues at day 6 post-infection for total pathology **(H)**, edema **(I)**, inflammation **(J)**, and epithelial injury **(K)** (n = 8-12 per group). **(L)** Quantification of submucosal edema as a percentage of total cecal tissue area. **(M)** Number of myeloperoxidase-positive (MPO^+^) inflammatory cells normalized to total tissue area per 20x field of view. **(N)** Representative H&E-stained cecal sections at six days post-infection. Blue outlined arrows indicate submucosal edema; orange outlined arrows indicate inflammatory cell infiltrates. **(O)** Representative MPO-stained cecal sections from each treatment group. Data points in panels (A-B) represent group means; data points in panels (D-M) represent individual biological replicates. Error bars indicate the standard error of the mean. * *P* < 0.05, ** *P* < 0.01., *** *P* < 0.001, **** **P* *< 0.0001.

Following antibiotic pre-treatment, mice were administered each mAb at 0.5 or 1 mg/kg (Fig 5A). Unlike infection with TcdB1^+^ or TcdB2^+^ strains (Figs 2 and 4), infection with M68 caused minimal clinical signs of disease. Mice did not develop diarrhea or exhibit signs of illness, such as lethargy, dehydration, or behavioral changes. Instead, they gained weight until days 2–3, followed by mild weight loss (Fig 5B). Mice treated with cIgG lost an average of 8% body weight between days 3–5 post-infection, a period during which colonization levels of M68 were comparable across all treatment groups (Fig 5C, 5D). TcdB3 levels in stool peaked on days 4–5, coinciding with the onset of weight loss (Fig 5C and 5E).

Gross pathological examination revealed differences in colon length and cecum area, both tissue-level markers of inflammation [11]. Colon lengths were significantly shorter in cIgG-treated mice compared to those treated with 1 mg/kg bezlotoxumab (**p* *= 0.0398; Fig 5F). Similarly, 1 mg/kg AZD5148 significantly reduced cecum shrinkage compared to cIgG (**p* *= 0.0165; Fig 5G).

To assess if the mild disease phenotype was specific to the M68 strain, we further characterized disease progression using three additional RT017 clinical isolates from IHMA: 2098716, 2103986, and 2111496 (S4 Fig and S1 Table). Mice infected with these strains also gained weight until day 3, followed by mild weight loss through day 6; however, M68-infected mice lost significantly more weight on day 6 than those infected with 2111496 (**p* *< 0.0001; S4A, S4B Fig). Peak *C. difficile* colonization occurred on day 3, coinciding with the onset of weight loss (S4C Fig). Colon length in mice infected with 2098716 or 2103986 was similar to that of healthy controls, whereas infection with 2111496 resulted in an average 1 cm increase in colon length (**p* *= 0.0307; S4D Fig). The only gross pathological sign of disease was significant cecum shrinkage in mice infected with 2098716 and 2103986 (**p* *= 0.0050 and 0.0040, respectively; S4E Fig). Assessment of histopathology revealed that the IHMA strains caused less severe edema and equally mild epithelial injury (S4F–S4J Fig). Intriguingly, the inflammation score was significantly higher in the ceca of 2098716-infected mice compared to M68 (**p* *= 0.0061; S4H Fig). Since M68-infected mice exhibited more severe weight loss, shrunken ceca, and high edema, we proceeded with M68 as a representative strain for RT017 infections.

Histopathological analysis of mAb-treated mice in the M68 infection model revealed minimal epithelial injury but pronounced submucosal edema (Fig 5H–5L and 5N). AZD5148 significantly reduced submucosal edema (**p* *= 0.0100 and 0.0230 for 0.5 and 1 mg/kg, respectively), while bezlotoxumab had a lesser effect (Fig 5I, 5L, and 5N). H&E-stained sections revealed an influx of polymorphonuclear cells into the submucosa (Fig 5N). Immunohistochemical staining of myeloperoxidase (MPO) showed that 1 mg/kg AZD5148 significantly reduced MPO^+^ cell infiltration to near healthy levels (*p *= 0.0031; Fig 5M–5O). In conclusion, while AZD5148 exhibited *p*oor neutralizing activity against TcdB3 *in vitro*, it still provided significant protection against edema, MPO^+^ cellular infiltration, and cecum shrinkage during M68 infection.

## Discussion

Prevention and treatment of primary and recurrent CDI remains a significant clinical challenge despite recent advancements in antibiotic therapies and live microbial therapeutics [1,14,15,29]. Bezlotoxumab (Zinplava), an anti-TcdB mAb, was approved in 2018 for the prevention of recurrent CDI in high-risk populations [16]; however, its recent discontinuation has left an unmet need for effective CDI therapies.

We have previously reported the broad neutralizing activity of another anti-TcdB mAb, AZD5148 (formerly PA41), against culture supernatants from diverse *C. difficile* strains *in vitro* [25,30]. Unlike bezlotoxumab, which binds the TcdB CROPS domain and prevents binding to the CSPG4 receptor [24,27], AZD5148 targets the TcdB GTD, blocking its translocation into host cells [25,26]. This mechanism of action downstream of surface receptor engagement could offer broader coverage across TcdB variants that differ in their receptor and substrate specificity. In this study, we present the first preclinical evidence of AZD5148 monotherapy providing protection in a CDI mouse model against three *C. difficile* strains expressing the most prevalent TcdB subtypes.

First, we evaluated the protective efficacy of AZD5148 in a mouse model of CDI using the epidemic-associated *C. difficile* strain R20291. AZD5148-treated mice were protected across a range of doses, while control mice exhibited significant weight loss and mortality. We next asked whether combining AZD5148 with the TcdA-neutralizing mAb, PA50, would provide greater protection against R20291 infection. While AZD5148 and the mAb combination reduced weight loss during acute infection, adding PA50 did not provide clinical benefit beyond that of AZD5148 alone. This aligns with studies conducted in a piglet model [31] and clinical trial data showing that the addition of actoxumab to bezlotoxumab did not enhance protection [16].

The efficacy of blocking TcdB in mice was confirmed using bezlotoxumab. However, while both anti-TcdB mAbs were protective against R20291 infection at 2.5 mg/kg, only AZD5148 maintained efficacy at lower doses. Interestingly, histological improvements were modest and limited to specific tissues, whereas systemic inflammatory markers, including CXCL1/KC and IL-6, were significantly reduced in serum. This suggests that, in mice, anti-TcdB mAbs may primarily act by neutralizing systemic toxin rather than preventing initial mucosal damage [32]. While there is currently no evidence of toxemia in human CDI, the efficacy of bezlotoxumab in mice correlates with the real-world efficacy of Zinplava in humans and suggests that the mouse model represents an informative step in pre-clinical testing [17].

Next, we compared the protective abilities of AZD5148 and bezlotoxumab in CDI induced by VPI 10463, a TcdB1-producing RT087 strain. AZD5148 prevented mortality and weight loss at 2.5 and 10 mg/kg doses, whereas bezlotoxumab only effectively prevented mortality at 10 mg/kg. Despite differences in survival, all groups showed severe intestinal pathology, again supporting a systemic mechanism of protection.

While AZD5148 appeared superior to bezlotoxumab in mitigating the effects of TcdB1 and TcdB2-producing strains, our *in vitro* neutralization data suggested that AZD5148 would be inferior to bezlotoxumab, and perhaps entirely ineffective, against the TcdB3-producing RT017 M68 strain. M68 was originally isolated from a multihospital CDI outbreak in Ireland [33], but a prior study characterized it as causing asymptomatic but persistent colonization in mice [34].

Consistent with this description, we observed minimal weight loss and changes in stool consistency, as well as no outward signs of severe infection, in mice infected with M68. To determine whether the mild disease phenotype was unique to M68, we evaluated three additional RT017 clinical isolates. The infected mice initially gained weight between days 1 and 3 post-infection, followed by a mild weight loss between days 3 and 5, a timing that coincided with peak TcdB3 levels in the stool of M68-infected mice. Tissue-level analyses painted a different picture of RT017 infection. Mice infected with M68, 2098716, or 2103986 exhibited signs of intestinal inflammation during CDI, characterized by smaller ceca. Histological examination revealed little to no epithelial damage but pronounced submucosal edema and inflammatory cell infiltrates. The similar patterns of weight gain followed by mild weight loss and limited pathological changes between RT017 strains supported our use of M68 for studying TcdB3-associated disease in mice.

With the model of M68 infection established, we assessed the protective properties of AZD5148 and bezlotoxumab against this TcdB3-producing strain. Treatment with 0.5 or 1 mg/kg AZD5148 significantly decreased edema severity, with the higher dose also reducing inflammatory MPO^+^ cell infiltrates to near-healthy levels. Surprisingly, bezlotoxumab did not provide significant protection against edema. Additionally, mice treated with cIgG and bezlotoxumab exhibited larger gut-associated lymphoid tissues, indicating a robust mucosal immune response to the infection [35].

Antibodies that neutralize toxin activity *in vitro* are expected to protect animals *in vivo* through a mechanism of direct toxin binding and neutralization. While it remains possible that AZD5148 or bezlotoxumab could bind the surface of *C. difficile*, we do not think that antibody-dependent cellular cytotoxicity and complement-dependent cytotoxicity represent significant mechanisms of protection. Consistent with this expectation, a previous study demonstrated that mutant versions of anti-toxin antibodies actoxumab and bezlotoxumab (N297Q), which cannot bind Fcγ receptors and complement initiator C1q, still provided robust protection in mice challenged with a TcdB2-producing strain [28]. Additionally, we did not observe a significant reduction in *C. difficile* colonization following antibody treatment. Effective opsonization would require that a substantial amount of the antibody has access to the colonic lumen—something we consider unlikely given the lack of protection against epithelial injury in IP mAb-treated mice.

The precise causes of severe sequelae in this mouse model of CDI remain unclear. In one scenario, the toxins gain access to the bloodstream and cause systemic intoxication and organ failure [32]. In another scenario, the tissue damage caused by toxins and the influx of neutrophils allows commensal organisms to gain access to the bloodstream, resulting in sepsis and multi-organ failure [36]. While the mechanism of death is unclear, we assume that an anti-toxin antibody will be most effective if it can block the initial events causing damage and inflammation in the tissue. While the histopathology scoring indicated a minimal impact on epithelial damage in the R20291- and VPI 10463-infected mice, it is notable that mice treated with AZD5148 and high doses of bezlotoxumab were largely protected from CDI-induced death. Further, there was a significant reduction in submucosal edema and MPO^+^ staining in the milder disease caused by M68 infection. These data suggest that some antibody reaches the tissue and exerts a protective effect. As suggested for bezlotoxumab, it is possible that mucosal injury may even be important for directing more AZD5148 from the bloodstream into the infected tissue.

For years, the *C. difficile* field has relied on immortalized cell line-based neutralization assays to evaluate the efficacy of therapeutic antibodies [6]. As our understanding of toxin receptors and cellular tropism has evolved, the question of what cell line should be used in neutralization assays has become more complex. For example, although AZD5148 and bezlotoxumab differed by four orders of magnitude in EC_50_ values in Vero cells, both provided similar levels of protection at 2.5 mg/kg in mice (Fig 3A and 3B). In this case, the lower EC_50_ of AZD5148 correlated with its ability to protect at doses as low as 0.5 mg/kg (Fig 3D and 3E). However, the dose-response relationship was not linear. Our data suggest that 0.1 mg/kg of AZD5148 is entirely sufficient to protect against TcdB2 in the R20291 infection model (S1 Fig). In contrast, higher doses were required for protection against VPI 10463 (Fig 4C and 4I), consistent with both the higher levels of toxins produced by this strain and the higher EC_50_ of AZD5148 against TcdB1.

The observation that AZD5148 protects a TcdB3-expressing strain *in vivo* is, at first glance, unexpected, given the lack of neutralization *in vitro*. However, there are two unique features of the TcdB3 sequence worth noting. The first is that the major sequence differences between TcdB3 and other TcdB subtypes are located in the GTD [19,20]. The effect of these differences is that, unlike most TcdB GTDs, which glucosylate Rho-family GTPases, the TcdB3 GTD is specific for Ras family GTPases [37,38]. While the impact of having different targets has not been defined *in vivo*, the toxins cause distinct morphological changes on cells *in vitro*. TcdB3-treated cells exhibit reduced adherence to plates [37]; therefore, the viability indicator used in standard toxin neutralization assays may not be well-suited for assessing the neutralizing potential of TcdB3-directed antibodies. The second feature is that, despite the significant sequence differences that impact the GTD substrate specificity, the epitope where AZD5148 binds is highly conserved [21]. The crystal structure of the TcdB GTD:AZD5148 Fab complex revealed only one amino acid contact residue that differed between TcdB3 and other subtype sequences; TcdB1 and TcdB2 have a tyrosine at the center of the AZD5148 epitope that is replaced with a histidine in TcdB3 [21,26].

In conclusion, our data show that AZD5148 provides robust protection in a mouse model of CDI caused by diverse *C. difficile* strains. By blocking the delivery of toxic cargo into the cell, AZD5148 efficacy is independent of the receptor tropism differences observed across TcdB subtypes. Further, the AZD5148 epitope is, for the most part, strictly conserved across TcdB subtypes. AZD5148 is currently being evaluated in a Phase I clinical trial for safety, tolerability, and pharmacokinetics in healthy adults (NCT06469151).

## Materials and methods

### Ethics statement

All animal procedures were approved by the Vanderbilt University Medical Center Institutional Animal Care and Use Committee (protocol M1700185-02) and adhered to ARRIVE guidelines. Mice were housed in an AAALAC-accredited facility under a 12-hour light/dark cycle with *ad libitum* access to food, water, and enrichment. Mouse health was monitored daily, and moribund animals were humanely euthanized by CO_2_ inhalation followed by cervical dislocation. All animals used in this study were C57BL/6J mice, 9–12 weeks of age, purchased from Jackson Laboratories. Mice were acclimated to the new facility for one week before antibiotic treatment. Cages were changed every two weeks to ensure clean bedding.

### Toxin purification

The TcdB2 and TcdB3 constructs were gifts from the laboratories of Jimmy Ballard and Hanping Feng, respectively. Recombinant TcdB1, TcdB2, and TcdB3 holotoxin constructs were expressed in *Bacillus megaterium* with C-terminal His_8_ tags and purified as previously described [26,39]. Purification involved Ni-affinity chromatography, followed by ion-exchange fractionation and size-exclusion chromatography. Proteins were eluted in a buffer comprised of 20 mM HEPES, pH 6.9, and 50 mM NaCl, and then sterile-filtered through a 0.2 µm membrane. Protein purity was confirmed by SDS-PAGE, and concentrations were determined by absorbance where TcdB1: ε_0.1%, 280 nm_, 1.067 M^-1^ cm^-1^, molecular weight, 269,170 Da; TcdB2: ε_0.1%, 280 nm_, 1.085 M^-1^ cm^-1^, molecular weight, 270,505 Da; TcdB3: ε_0.1%, 280 nm_, 1.057 M^-1^ cm^-1^, molecular weight 269,324 Da. Plasmid constructs are listed in S2 Table.

### Monoclonal antibodies

Anti-TcdB mAbs AZD5148* (formerly PA41) and bezlotoxumab (formerly CDB1), and anti-TcdA mAb PA50 were expressed in CHO-derived cell lines and purified by protein A chromatography [25,40]. The control mAb (cIgG) R347 is an anti-HIV gp120 [41].

*Note: the AZD5148 mAb used in this study is different from the AZD5148 used in the clinical trial NCT06469151. The clinical trial AZD5148 features a M252Y/S254T/T256E (YTE) substitution in the Fc portion, designed to extend the half-life of the mAb in humans.

### *In vitro* toxin neutralization assays

The neutralizing activities of AZD5148 and bezlotoxumab mAbs against the three TcdB subtypes were determined in a cytotoxic assay with Vero cells as previously described [42]. Briefly, Vero cells (ATCC, CCL-81, Manassas, VA, USA) were seeded at 1,500 cells/well in 96-well black clear bottom plates (Corning Inc., Corning, NY, USA) in 100 µl of Eagle’s Minimum Essential Medium supplemented with 10% fetal bovine serum (Corning Inc.) and incubated overnight at 37 °C with 5% CO_2_. The next day, serial dilutions of mAbs were pre-incubated with 30 fM purified TcdB1, TcdB2, or TcdB3 for 30 minutes at room temperature. Media was aspirated, 90 µL of fresh media was added, and then 10 µL of the mixture was transferred to the cells. Following 72 hours of incubation, the media was aspirated, and fresh media (100 µL) plus 20 µl/well of CellTiter Blue (Promega Corp., Madison, WI) was added to the cells. After 3.5 hours of incubation, the fluorescence was measured at optical density (OD)_560/590nm_ excitation/emission with a BioTek Cytation 5 plate reader (Agilent Technologies Inc., Santa Clara, CA, USA). Cell viability was normalized within each plate ((OD toxin-OD mAb signal – OD media-only signal)/ (OD toxin-only signal), and EC_50_ was calculated in GraphPad Prism v10.2.3. using the least squares fit of the log(agonist) vs. response -- variable slope (four parameters) model.

### Bacterial strains and spore preparation

*Clostridioides difficile* strains R20291 and M68 were gifts from the laboratories of Sarah Kuehne and Robert Fagan, respectively. VPI 10463 was acquired from ATCC, and RT017 strains 2098716, 2103986, and 2111496 were acquired from International Health Management Associates (IHMA) (S1 Table). Strains were cultured at 37 °C on brain-heart-infusion media plates supplemented with 0.5% yeast extract, 0.1% cysteine, and 0.1% taurocholate (BHIS-TA) in an anaerobic chamber (90% N_2_, 5% H_2_, 5% CO_2_). For *in vivo* infections, individual colonies were transferred from BHIS-TA plates into 4 mL BHIS and incubated overnight at 37 °C anaerobically. The next day, the culture was inoculated into 46 mL of Clospore media [43] and cultured for ten days. Cultures were then centrifuged at 4,000 x g at 4 °C, and pellets were washed three times in cold, sterile water. Spores were then suspended in 1 mL sterile PBS and heat-treated at 65 °C for 20 minutes to kill vegetative cells. Viable spores were enumerated through serial dilutions and plating on BHIS-TA plates. Spore stocks were stored at 4 °C until use, and strains were stored long-term at -80 °C in 20% glycerol.

### Mouse infection model

Protective efficacies of mAbs were tested in a mouse CDI model as described [11,44]. Nine-week-old C57BL/6J mice were treated with cefoperazone (0.5 mg/mL) in drinking water *ad libitum* for five days, followed by two days of regular water. Mice were then anesthetized by isoflurane inhalation and injected intraperitoneally with mAbs diluted in sterile PBS to a final volume of 100 µl 24 hours before infection. Infections were performed via oral gavage with 1,000 spores of R20291 (TcdB2), VPI 10463 (TcdB1), M68 (TcdB3), 2098716 (TcdB3), 2103986 (TcdB3), or 2111496 (TcdB3) in 100 µl of sterile PBS.

Mice were monitored daily for weight, body condition, and stool consistency. On indicated days, bacterial burden was quantified by macerating stool in sterile PBS and dilution plating on taurocholate-cycloserine-cefoxitin-fructose agar (TCCFA) semi-selective medium comprising 0.1% (w/v) TA, 250 µg/mL D-cycloserine, 16 µg/mL cefoxitin, 0.6% (w/v) fructose, 40 mg/mL Proteose Peptone No. 2 (Thermo Fisher Scientific, Waltam, MA, USA), 5 mg/mL Na_2_HPO_4_, 1 mg/mL KH_2_PO_4_, 2 mg/mL NaCl, 0.1 mg/mL MgSO_4_ anhydrous, and 20 mg/mL agar. Mice were euthanized if they lost >20% of their initial body weight or showed signs of morbidity.

Mice were age- and sex-matched where possible and randomly assigned to treatment groups. Sample sizes were determined *a priori* using power analysis (effect size = 0.5, α = 0.05, power = 80%) based on prior CDI mouse model data.

### Histopathology and immunohistochemistry

Ceca and colons were collected post-mortem, measured, flushed with PBS, and fixed in 10% neutral buffered formalin (NBF) at room temp for 24 hours. Tissues were embedded in paraffin, sectioned (5 µm), and stained with hematoxylin and eosin (H&E). A board-certified gastrointestinal pathologist, blinded to treatment groups, scored edema, inflammation, and epithelial injury based on previously established criteria [11,44]. Representative images were captured using a BioTek Cytation 5 plate reader with 1.25x and 10x objectives.

Submucosal edema was quantified using AI-driven image segmentation in QuPath v0.5.1 [45,46]. The area of edematous tissue was segmented and divided by the total area of the cecum segment in two separate cecum corpus segments per mouse [47].

To quantify MPO^+^ cell infiltrates, five-micron sections were deparaffinized in xylenes and rehydrated through an ethanol gradient before antigen retrieval in 10 mM sodium citrate, pH 6.0, with 0.05% Tween-20 for 15 minutes in a pressure cooker. Tissues were blocked with BLOXALL (Vector Laboratories, Newark, CA, USA), then stained with anti-myeloperoxidase antibody EPR20257 (Abcam Limited, Cambridge, United Kingdom) diluted 1:500 overnight at 4 °C. Staining and development were performed with the VECTASTAIN Elite ABC Kit, Peroxidase (Rabbit IgG) (Vector Laboratories) and ImmPACT DAB Substrate Kit, Peroxidase (Vector Laboratories) following the manufacturer’s instructions. MPO^+^ cells were quantified in QuPath v0.5.1 and normalized to the total tissue area in the image in two separate cecum corpus segments per mouse.

### Cytokine analyses

Forty-eight hours post-infection with R20291, mice were euthanized, blood collected via cardiac puncture and transferred into serum separating tubes (Sarstedt, Nümbrecht, Germany). Sera was separated by centrifugation at 5,000 x g for 10 minutes at 4 °C and stored at -80 °C until use. A small snip of the cecum apex and distal colon was collected during necropsies, rinsed in PBS, then frozen in liquid N_2_ and stored at -80 °C until use. Pro-inflammatory cytokines were measured with a MesoScale 10-V Plex pro-inflammatory mouse cytokines kit (MesoScale, Gaithersburg, MD, USA).

### Toxin measurement

TcdB3 levels in stool were quantified using a nanobody-based ELISA as previously described [42]. Stool was diluted to 0.5 mg/mL in PBS with 2% bovine serum albumin, and toxin concentrations were interpolated from a standard curve of recombinant TcdB3.

### Statistical analysis

Data were analyzed and graphed using GraphPad Prism v10.6.0. Two-way ANOVA with Tukey’s post-hoc test was used for comparisons between multiple groups. Survival curves were analyzed using the log-rank (Mantel-Cox) test. Differences between two groups were considered statistically different if the p-value was less than 0.05.

## Supporting information

S1 Table Strains used in this study. (DOCX)

S2 Table Plasmids used in this study. (DOCX)

S1 Fig Individual body weight values from Fig 2. **(A)** Percent body weight relative to baseline in individual mice pre-treated with AZD5148 and infected with *C. difficile* R20291. The data correspond to the groups shown in Fig 2B. Each point represents a single animal; crossbars indicate the group median. **(B)** Percent body weight in individual mice pre-treated with AZD5148 or a combination of AZD5148 and PA50 prior to R20291 infection. The data correspond to the groups shown in Fig 2D. *P < 0.05, ** P < 0.01, *** P < 0.001.
(PDF)

S2 Fig Individual body weight data from Fig 3. Percent body weight relative to baseline in individual mice pre-treated with cIgG, AZD5148, or bezlotoxumab at **(A)** 2.5 mg/kg, **(B)** 1.25 mg/kg, or **(C)** 0.5 mg/kg, followed by infection with *C. difficile* R20291 (n = 8 per group). Each point represents a single animal; crossbars indicate the group median. * *P *< 0.05, ** *P *< 0.01, *** *P* < 0.001, **** *P *< 0.0001.
(PDF)

S3 Fig AZD5148 and Bezlotoxumab have minimal impact on tissue histopathology scores but reduce systemic markers of inflammation in response to R20291 infection. Histopathological scores for edema, inflammation, and epithelial injury were assessed in the ceca **(A-D)** or colons **(E-H)** of *C. difficile* R20291-infected mice (n = 5–6 per group) by a board-certified gastrointestinal pathologist blinded to treatment groups. **(I)** Representative H&E-stained images of ceca and colons collected two days post-infection. Edema is indicated by blue double arrows, inflammation by orange arrows, and epithelial injury by magenta arrows. **(J)** *C. difficile* R20291 colonization burden in shed stool (n = 6–12 per group). **(L-K)** Levels of inflammatory markers CXCL1/KC **(L)** and IL-6 **(K)** in serum, cecal, and distal colon tissues from infected or healthy control mice at two days post-infection (n = 5–10). * *P* < 0.05, ** *P* < 0.01., *** *P* < 0.001, **** *P *< 0.0001.
(PDF)

S4 Fig Infection with epidemic RT017 strains recapitulates mild disease phenotypes associated with M68 infection. **(A-B)** Percent body weight relative to baseline in mice infected with three *C. difficile* RT017 isolates from hospital outbreaks (n = 3 per IHMA strain or 17 for M68). **(C)** Daily *C. difficile* colonization burden in naturally passed stool (n = 3 per IHMA strain or 12 for M68). **(D)** Colon length and **(E)** cecum area measured at day 6 post-infection (n = 3 per IHMA strain, 7 for M68, or 5 for vehicle). **(F-I)** Histopathological scoring of the cecum for edema **(G)**, inflammation **(H)**, and epithelial injury **(I)** were performed by a gastrointestinal pathologist blinded to the conditions. **(J)** Representative H&E images of ceca on day six post-infection with IHMA strains. Orange arrows denote inflammatory cell infiltrates, blue highlights submucosal edema, and grey points to epithelial injury (apoptotic body). The M68 infection data used in this figure are the same as in Fig 5. Data points in panels (A, C-I) represent individual biological replicates; data points in panel B represent group means. Error bars indicate the standard error of the mean. * *P* < 0.05, ** *P* < 0.01.
(PDF)
