# Supplementary material for: The monoclonal antibody AZD5148 confers broad protection against TcdB-diverse Clostridioides difficile strains in mice
Source: PLoS Pathog. 2025 Nov 3;21(11):e1013651. doi: 10.1371/journal.ppat.1013651 (PMC12594360; doi:10.1371/journal.ppat.1013651)
Supplement: S1 Table — (DOCX) [file ppat.1013651.s001.docx]

**SUPPLEMENTAL TABLES**

| S1 Table. Strains used in this study | | | |  |
| --- | --- | --- | --- | --- |
| **Strain Name** | **PCR Ribotype** | **Toxins** | **Source** | **Reference** |
| R20291 | 027 | TcdA+ TcdB2+ CDT+ | Dr. Sarah Kuehne | [11,13] |
| VPI 10463 | 087 | TcdA+ TcdB1+ CDT- | ATCC |  |
| M68 | 017 | TcdA- TcdB3+ CDT- | Dr. Robert Fagan | [33] |
| 2098716 | 017 | TcdA- TcdB3+ CDT- | IHMA | This study |
| 2103986 | 017 | TcdA- TcdB3+ CDT- | IHMA | This study |
| 2111496 | 017 | TcdA- TcdB3+ CDT- | IHMA | This study |
