# Supplementary material for: The monoclonal antibody AZD5148 confers broad protection against TcdB-diverse Clostridioides difficile strains in mice
Source: PLoS Pathog. 2025 Nov 3;21(11):e1013651. doi: 10.1371/journal.ppat.1013651 (PMC12594360; doi:10.1371/journal.ppat.1013651)
Supplement: S2 Table — (DOCX) [file ppat.1013651.s002.docx]

| S2 Table. Plasmids used in this study | | | |
| --- | --- | --- | --- |
| **Number** | **Plasmid** | **Relevant characteristics** | **Source** |
| pBL377 | pC-His1622-*tcdB*1 | pC-HIS1622, TcdB_1-2366_, strain VPI10463 | Lab stock |
| pBL598 | pC-His1622-*tcdB*2 | pC-HIS1622, TcdB_1-2366_, strain R20291 | Lab stock |
| pBL1316 | pC-His1622-*tcdB*3 | pC-HIS1622, TcdB_1-2367_, strain M68 | Lab stock |
