# Supplementary figures and images for: The monoclonal antibody AZD5148 confers broad protection against TcdB-diverse Clostridioides difficile strains in mice
Source: PLoS Pathog. 2025 Nov 3;21(11):e1013651. doi: 10.1371/journal.ppat.1013651 (PMC12594360; doi:10.1371/journal.ppat.1013651)

**A**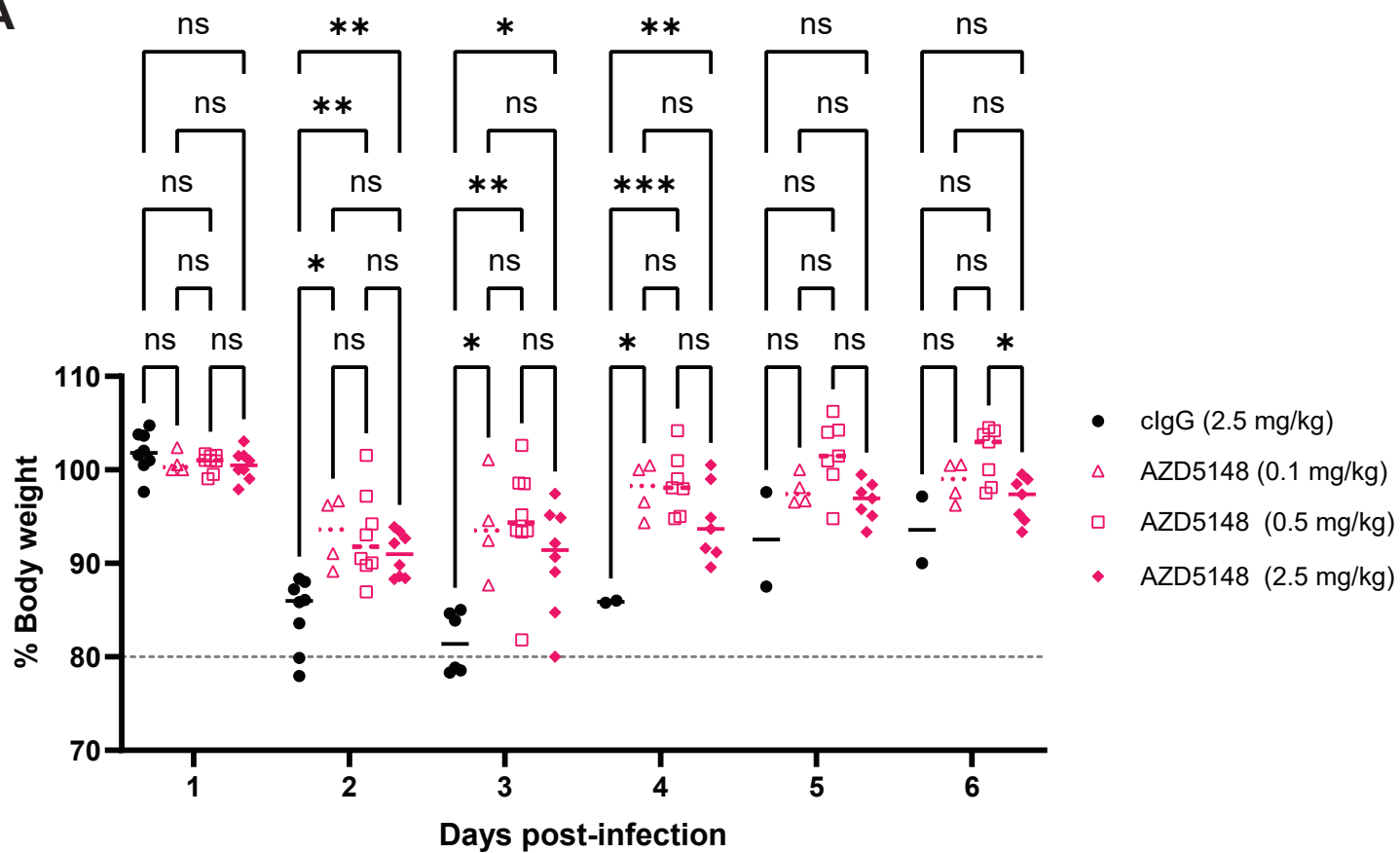**B**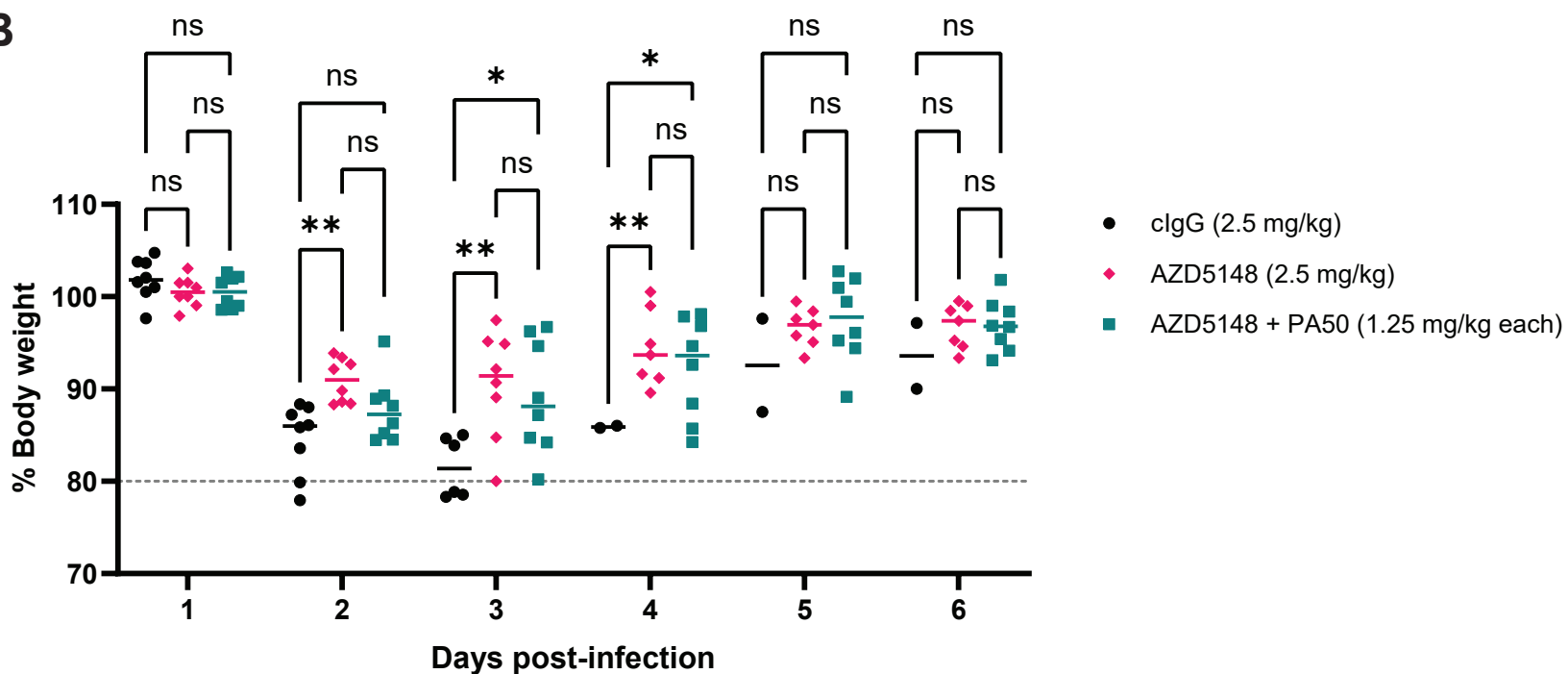

Supplement: S1 Fig — (A) Percent body weight relative to baseline in individual mice pre-treated with AZD5148 and infected with C. difficile R20291. The data correspond to the groups shown in Fig 2B. Each point represents a single animal; crossbars indicate the group median. (B) Percent body weight in individual mice pre-treated with AZD5148 or a combination of AZD5148 and PA50 prior to R20291 infection. The data correspond to the groups shown in Fig 2D. *P < 0.05, ** P < 0.01, *** P < 0.001. (PDF) [file ppat.1013651.s003.pdf]

**A**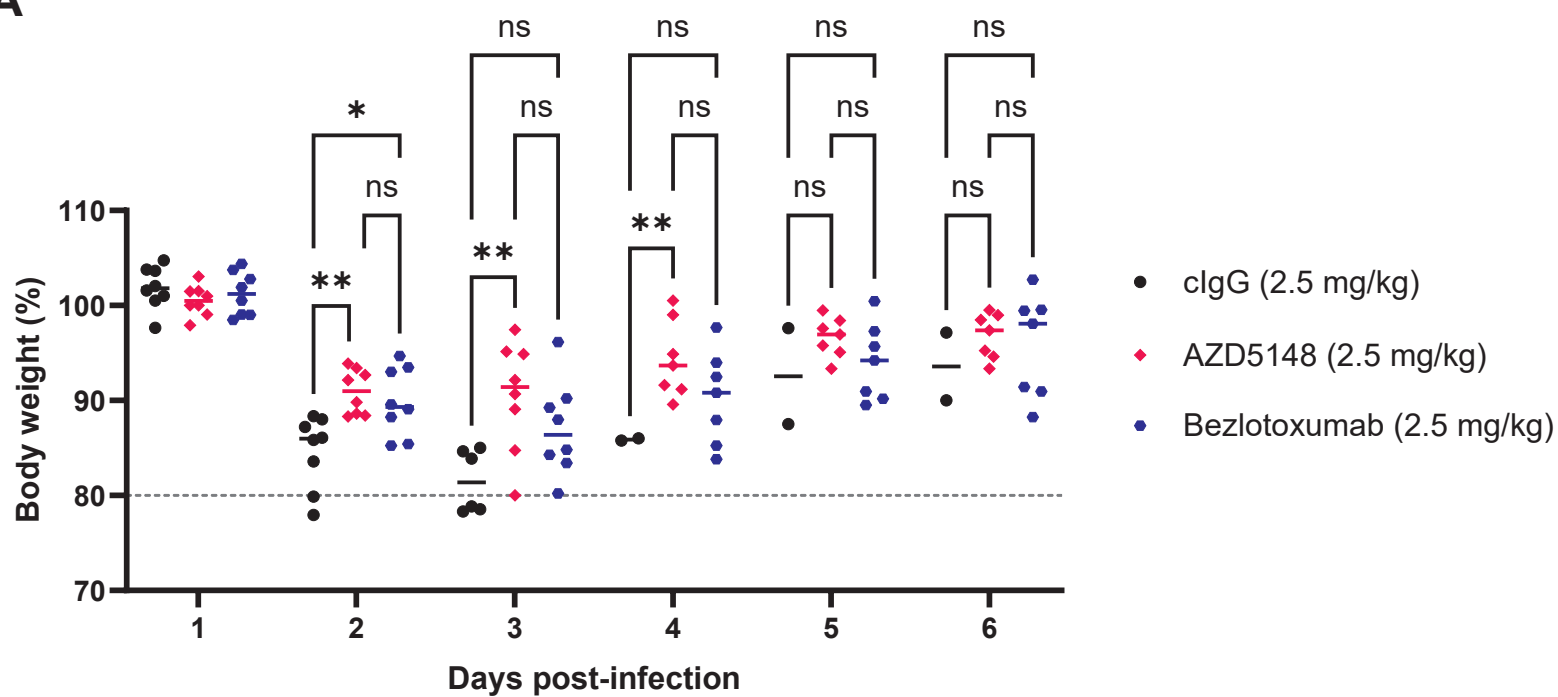**B**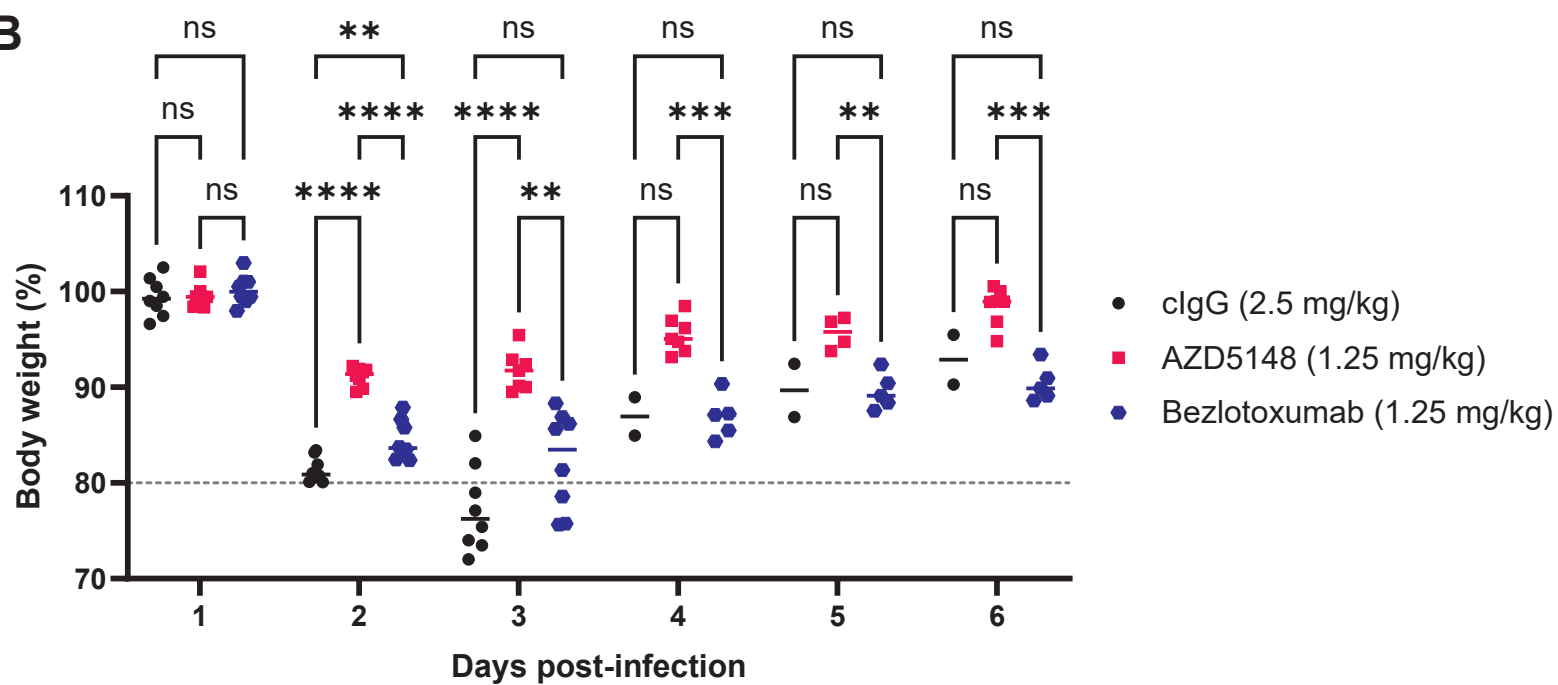**C**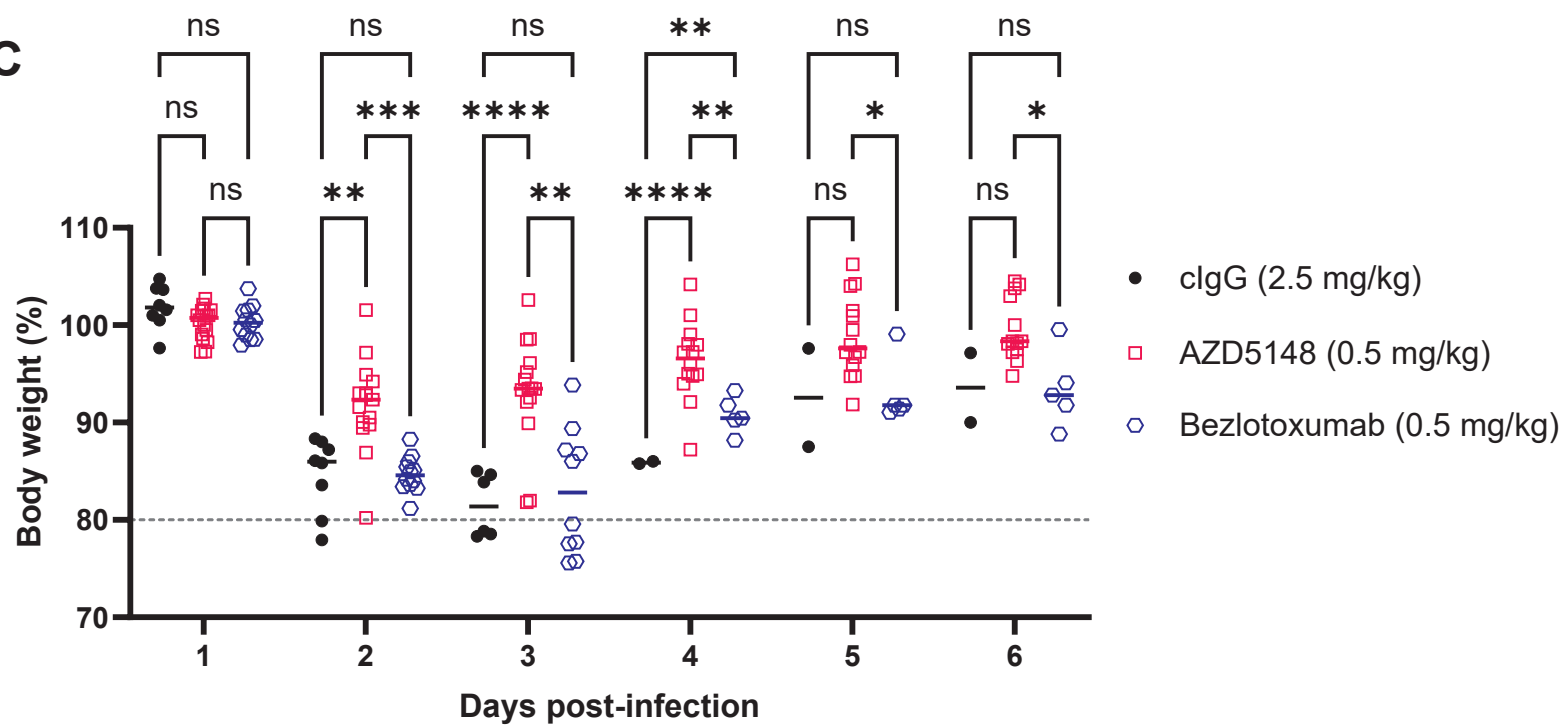

Supplement: S2 Fig — Percent body weight relative to baseline in individual mice pre-treated with cIgG, AZD5148, or bezlotoxumab at (A) 2.5 mg/kg, (B) 1.25 mg/kg, or (C) 0.5 mg/kg, followed by infection with C. difficile R20291 (n = 8 per group). Each point represents a single animal; crossbars indicate the group median. * P < 0.05, ** P < 0.01, *** P < 0.001, **** P < 0.0001. (PDF) [file ppat.1013651.s004.pdf]

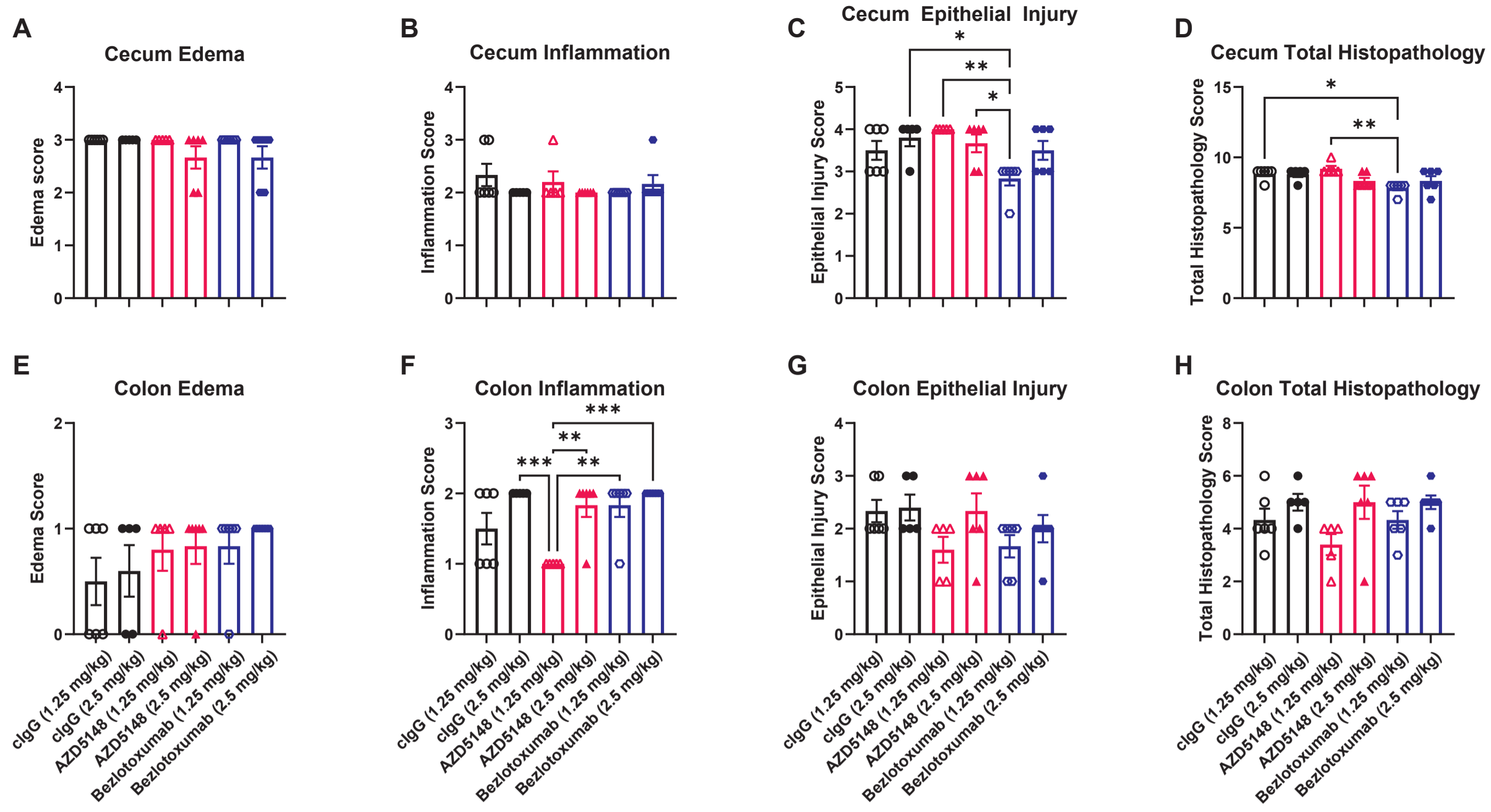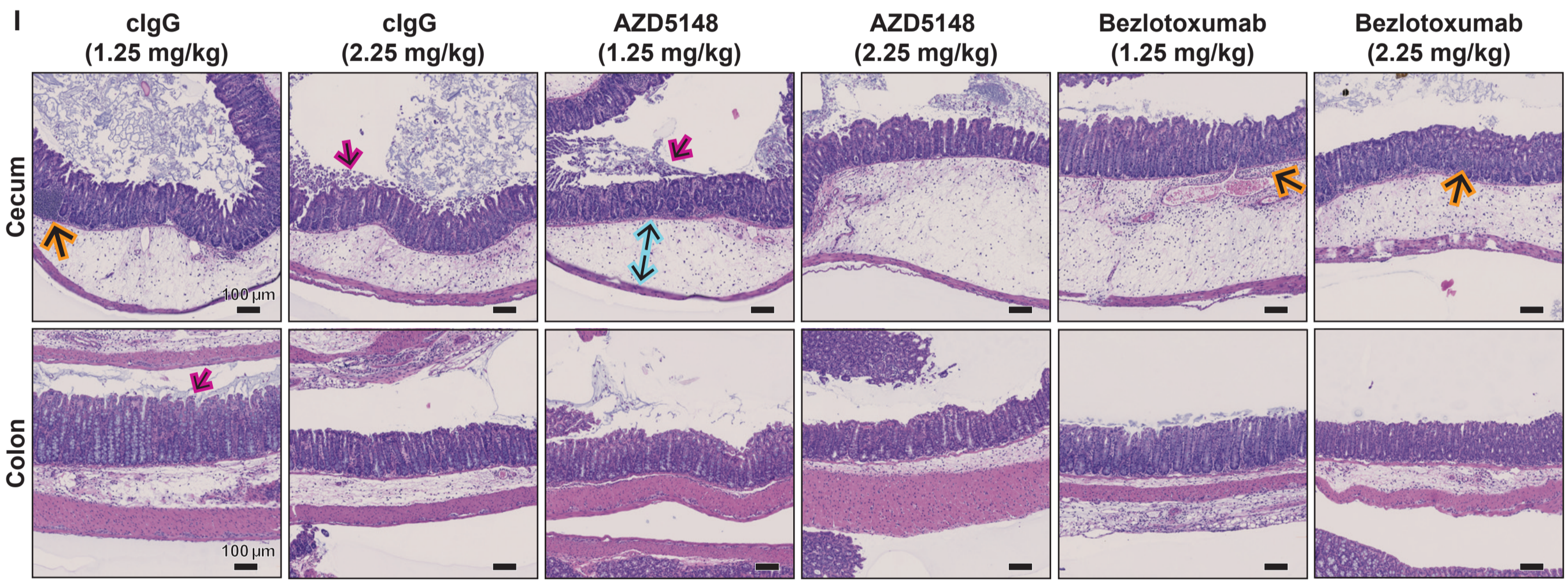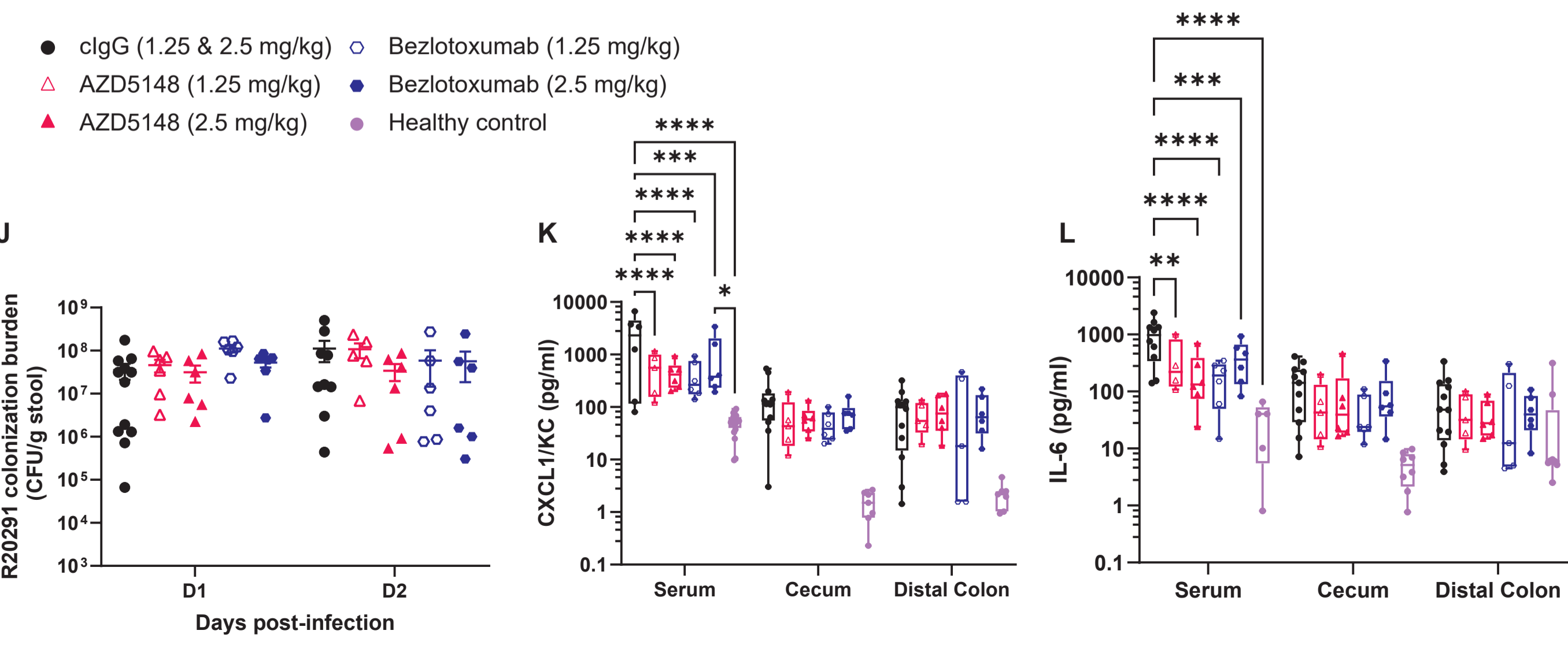

Supplement: S3 Fig — Histopathological scores for edema, inflammation, and epithelial injury were assessed in the ceca (A-D) or colons (E-H) of C. difficile R20291-infected mice (n = 5–6 per group) by a board-certified gastrointestinal pathologist blinded to treatment groups. (I) Representative H&E-stained images of ceca and colons collected two days post-infection. Edema is indicated by blue double arrows, inflammation by orange arrows, and epithelial injury by magenta arrows. (J) C. difficile R20291 colonization burden in shed stool (n = 6–12 per group). (L-K) Levels of inflammatory markers CXCL1/KC (L) and IL-6 (K) in serum, cecal, and distal colon tissues from infected or healthy control mice at two days post-infection (n = 5–10). * P < 0.05, ** P < 0.01., *** P < 0.001, **** P < 0.0001. (PDF) [file ppat.1013651.s005.pdf]
