# Supplementary material for: The monoclonal antibody AZD5148 confers broad protection against TcdB-diverse Clostridioides difficile strains in mice
Source: PLoS Pathog. 2025 Nov 3;21(11):e1013651. doi: 10.1371/journal.ppat.1013651 (PMC12594360; doi:10.1371/journal.ppat.1013651)
Supplement: S4 Fig — (A-B) Percent body weight relative to baseline in mice infected with three C. difficile RT017 isolates from hospital outbreaks (n = 3 per IHMA strain or 17 for M68). (C) Daily C. difficile colonization burden in naturally passed stool (n = 3 per IHMA strain or 12 for M68). (D) Colon length and (E) cecum area measured at day 6 post-infection (n = 3 per IHMA strain, 7 for M68, or 5 for vehicle). (F-I) Histopathological scoring of the cecum for edema (G), inflammation (H), and epithelial injury (I) were performed by a gastrointestinal pathologist blinded to the conditions. (J) Representative H&E images of ceca on day six post-infection with IHMA strains. Orange arrows denote inflammatory cell infiltrates, blue highlights submucosal edema, and grey points to epithelial injury (apoptotic body). The M68 infection data used in this figure are the same as in Fig 5. Data points in panels (A, C-I) represent individual biological replicates; data points in panel B represent group means. Error bars indicate the standard error of the mean. * P < 0.05, ** P < 0.01. (PDF) [file ppat.1013651.s006.pdf]

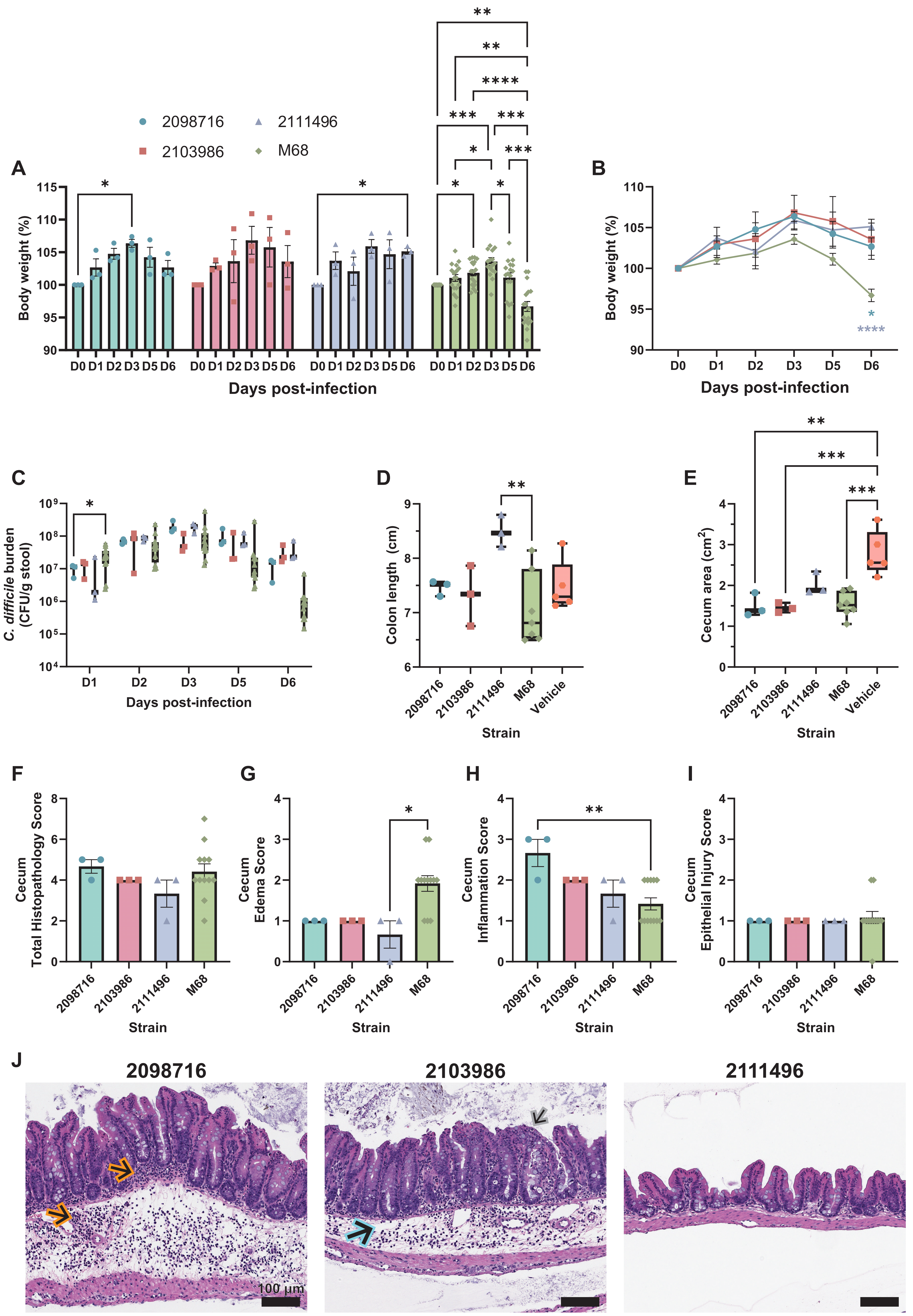
A
B
C
D
E
F
G
H
I
J
2098716
2103986
2111496
M68
Body weight (%)
Days post-infection
D0 D1 D2 D3 D5 D6
C. difficile burden (CFU/g stool)
Colon length (cm)
Cecum area (cm²)
Strain
Vehicle
Cecum Total Histopathology Score
Cecum Edema Score
Cecum Inflammation Score
Cecum Epithelial Injury Score
100 µm
